# Safety and Efficacy of rhBMP-2 for Treating Acute Traumatic Fractures of the Upper and Lower Extremities: A Multicenter Prospective Study

**DOI:** 10.3390/jcm15031176

**Published:** 2026-02-03

**Authors:** Seungyeob Sakong, Seokjun Hong, Wonseok Choi, Seonghyun Kang, Jae-Woo Cho, Whee Sung Son, Jeong-Seok Choi, Chang-Jin Yon, Won-Tae Cho, Jong-Keon Oh

**Affiliations:** 1 Department of Orthopaedic Surgery, Ajou University Hospital, School of Medicine, Ajou University, Suwon 16499, Republic of Korea; 2 Department of Orthopaedic Surgery, Korea University Guro Hospital, College of Medicine, Korea University, Seoul 03080, Republic of Korea; 3 Department of Orthopaedic Surgery, Yeungnam University Medical Center, College of Medicine, Yeungnam University, Daegu 42415, Republic of Korea; 4 Department of Orthopaedic Surgery, Armed Forces Capital Hospital, Seongnam 13574, Republic of Korea; 5 Department of Orthopaedic Surgery, Keimyung University Dongsan Hospital, School of Medicine, Keimyung University, Daegu 42601, Republic of Korea

**Keywords:** bone morphogenetic protein-2, hydroxyapatite, fracture, trauma, bone regeneration

## Abstract

**Background:** Delayed or non-union fractures comprise 5–10% of cases, indicating the need for biologic interventions. Recombinant human bone morphogenetic protein-2 (rhBMP-2) is a potent osteoinductive agent; yet, collagen carrier-based uncontrolled release causes adverse events. We evaluated the safety and efficacy of a hydroxyapatite (HA) carrier-based rhBMP-2 delivery system for acute traumatic upper and lower fractures exhibiting bone defects. **Methods:** This prospective, multicenter, single-arm clinical trial enrolled 90 patients who underwent surgery using a hydroxyapatite (HA) carrier-based rhBMP-2 delivery system (Novosis^TM^). Radiographically validated union at 6 and 12 months post-surgery and treatment success (union without additional surgery) were used to assess efficacy. The incidence, type, and severity of all device-related adverse events during follow-up were monitored by investigators to evaluate safety. **Results:** Of the 90 patients enrolled, 81 were included in the full analysis set. The mean age was 58.5 years, and 18.6% (15/81) had open fractures. At 6 months post-surgery, radiographically validated union was achieved in 81.5% (66/81) of patients, increasing to 96.2% (77/81) at 12 months after surgery. Treatment success was 95.0% (76/81). Adverse events were rare (1/81, 1.2%). No ectopic ossification, systemic complications, or severe inflammatory responses were observed. **Conclusions**: HA-based rhBMP-2 intervention demonstrated favorable union rates and safety with minimal complications in acute upper and lower fractures with bone defects. The biocompatibility and controlled-release properties of HA likely improved efficacy and reduced complications. Results should be interpreted as feasibility data from a heterogeneous case series without a control group. Larger randomized controlled comparative trials are warranted for optimal dosing and evaluating efficacy and cost-effectiveness.

## 1. Introduction

Fracture healing is a complex biological process, and despite technological advancements in surgical methods, approximately 5–10% of fractures fail to heal appropriately, resulting in delayed union or non-union [1]. These complications present persistent therapeutic challenges and often result in long-term functional impairment and reduced quality of life. Consequently, there is a growing need to enhance conventional mechanical fixation with biologic strategies to accelerate osteogenesis and improve outcomes [2].

Autologous bone grafting remains the standard method for promoting osteogenesis in complex fractures [3]. However, it carries notable drawbacks, including limited graft availability, donor site morbidity, and inconsistent biological effectiveness [4,5]. In recent years, recombinant human Bone Morphogenetic Protein-2 (rhBMP-2) has emerged as a promising biologic alternative to traditional grafting supported by its potential to accelerate fracture healing and reduce the complications associated with autografts [6,7,8,9,10].

The discovery of BMPs marked a pivotal advancement in our understanding of fracture healing biology. Among them, rhBMP-2, which is a member of the transforming growth factor-beta (TGF-β) superfamily, has demonstrated strong osteoinductive properties. It promotes bone formation by upregulating osteogenic gene expression in mesenchymal stem cells, thereby inducing their differentiation into osteoblasts [11,12]. Preclinical in vitro and in vivo studies have confirmed the biological activity and safety profile of rhBMP-2. Clinical trials in humans have further explored its optimal dosage, carrier systems, and clinical efficacy, especially in the management of open fractures [13,14,15,16,17,18], non-unions [19,20,21,22,23,24,25,26], and critical-sized bone defects [27,28].

Despite its proven osteoinductive potential, the clinical use of rhBMP-2 with absorbable collagen sponge (ACS) carriers has been linked to adverse effects, including soft tissue swelling, ectopic bone formation, and premature osteolysis, attributed to the burst-release profile and uncontrolled local concentrations of the protein [29,30,31,32].

Novosis^TM^, a hydroxyapatite (HA) carrier-based rhBMP-2 delivery system, has been developed to overcome these challenges. The HA matrix utilizes a specialized dual-porosity architecture, composed of macropores (100–1000 μm) for angiogenesis and interconnected micropores for protein adsorption, to provide a superior scaffold for bone regeneration. Unlike traditional absorbable collagen sponges (ACSs), which often exhibit rapid “burst release” that discharges nearly 100% of the rhBMP-2 within 24 h, this design leverages HA’s natural non-covalent binding sites to decelerate release over several weeks. By maintaining approximately 80% of the loaded protein after the first week, the matrix ensures localized osteoinductive potency and provides a sustained-release mechanism that supports all sequential phases of bone repair. This prolonged action effectively mitigates dose-dependent complications, such as massive soft-tissue swelling or ectopic bone formation, and promotes the development of high-quality trabecular bone rather than the fatty marrow often associated with uncontrolled release profiles [33,34,35,36].

This prospective, multicenter clinical trial aimed to assess the efficacy and safety of Novosis^TM^ in patients with acute upper or lower extremity fractures requiring bone grafting. The primary endpoint was radiographic validation of union at 6 and 12 months postoperatively. Secondary endpoints included evaluation of adverse events related to the intervention.

## 2. Patients and Methods

### 2.1. Study Design

We carried out a prospective, multicenter, clinical trial with a single-arm study design between September 2022 and December 2024 to evaluate the efficacy and safety of Novosis^®^ (CGBio Inc., Seongnam, Republic of Korea): an HA carrier-based rhBMP-2 delivery system for treating patients with acute traumatic fractures of the upper and lower extremities.

Patients were initially assessed for eligibility at the time of arrival at the hospital on the basis of selection criteria derived from the study protocol, which had been approved by the Korean Ministry of Food and Drug Safety. Inclusion criteria were as follows: (i) patients aged over 18 years at the time of injury with radiographic evidence of epiphyseal closure; (ii) patients diagnosed with acute traumatic lower and upper extremity fractures requiring operative treatment by internal fixation; and (iii) a traumatic bone defect measuring less than 5 cm in length and involving at least 50% of the circumference of the diaphysis which is clinically considered as a standard of care for bone graft [3,37,38]. The exclusion criteria were as follows: (i) patients who were pregnant or breastfeeding a child; (ii) patients who were fertile women and did not agree to maintain contraception for 1 year; (iii) patients with an active malignant tumor; (iv) patients with a possibility of metastasis from remission; (v) presence of infection on injured site before surgical treatment; (vi) known hypersensitivity to HA or rhBMP-2; (vii) inability to complete scheduled follow-up visits; and (viii) uncontrolled diabetes or coagulation disorders.

### 2.2. Efficacy Assessments

The participants were evaluated at four predetermined study visits: screening (Visit 1), surgery day (Visit 2), and at 6-month (Visit 3) and 12-month (Visit 4) postoperative intervals. At each visit, demographic and clinical information, surgical details, and concomitant medications were documented. Radiographic evaluations were performed at 6 and 12 months postoperatively, with standard anteroposterior and lateral radiographs acquired to assess progress in bone healing. Any additional surgical interventions required to promote fracture union and all adverse events (AEs) were recorded throughout the follow-up period.

The primary outcome was the bone union rate at 12 months postoperatively. The secondary outcome endpoints were as follows: (i) the bone union rate at 6 months post-surgery, (ii) the treatment success rate, defined as achieving fracture union without requiring additional surgical intervention, and (iii) the incidence of additional surgical procedures conducted to facilitate fracture union. Radiographically validated bone union was defined with a RUST score of ≥9 [39]. Non-union was defined as a fracture that had not healed for at least 9 months with no signs of healing for three consecutive months. As a separate assessment of efficacy for radiographically validated fracture healing, an independent evaluation of serial radiographs was conducted by two fellowship-trained orthopedic trauma surgeons (W.C and S.K), each with more than 10 years of clinical experience in trauma management. The disagreements were adjudicated by a third reviewer. The disagreements were adjudicated by a third reviewer.

### 2.3. Safety Assessments

The safety outcomes included the incidence, type, severity, and seriousness of adverse events occurring during the study period. All adverse events were systematically monitored by all participating investigators and classified according to severity (mild, moderate, severe), seriousness (serious or non-serious), and relationship to the investigational device (definitely related, probably related, possibly related, possibly not related, definitely not related, unknown) by all participating investigators, with final adjudication of seriousness and causality determined by the principal investigator (J.K.O). Anticipated device-related adverse events of interest (AEI) included inflammation, infection, hardware failure, pain, swelling, seroma, ectopic bone formation, and osteolysis. Fracture-related infection (FRI) was diagnosed according to the criteria established by the FRI Consensus Group, as described by Metsemakers et al. [40]. All adverse events were coded and categorized using the Medical Dictionary for Regulatory Activities.

### 2.4. Intervention

**Figure 1:**
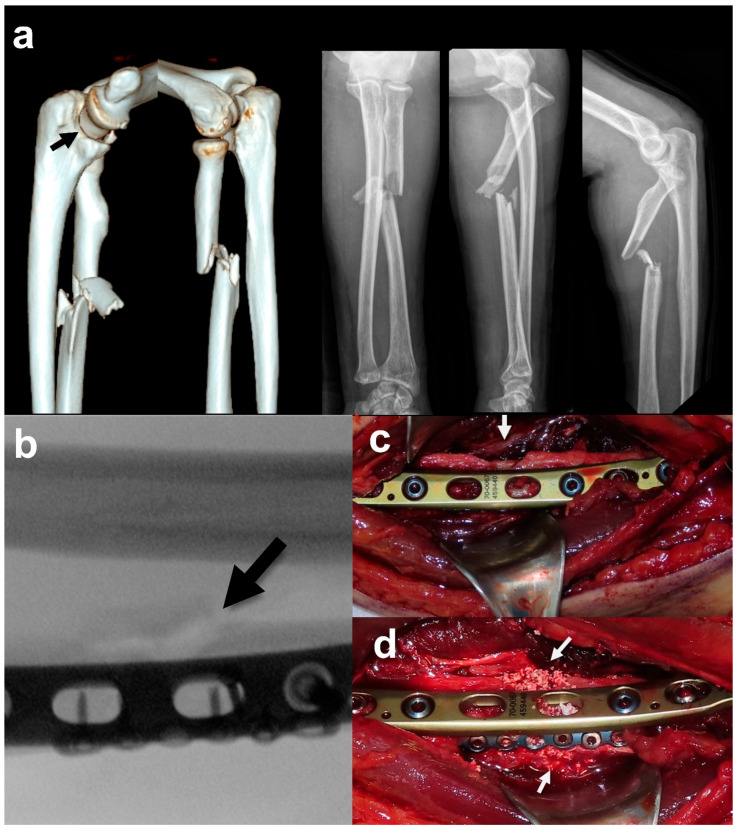

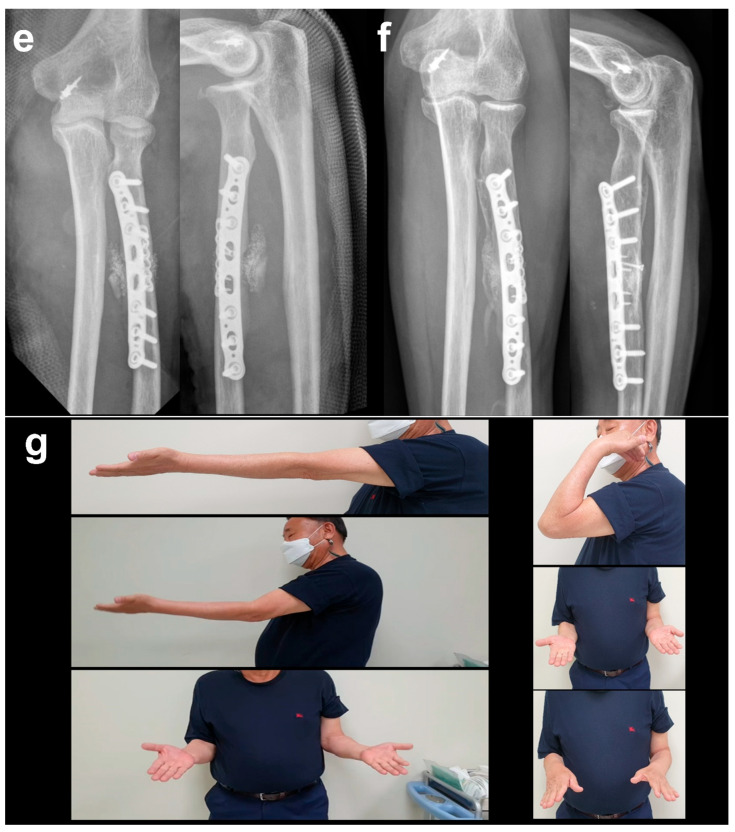
Surgical implementation of hydroxyapatite-carrier rhBMP-2. (**a**) A 69-year-old male patient diagnosed with Arbeitsgemeinschaft für Osteosynthesefragen/Orthopaedic Trauma Association (AO/OTA) 2R2-B3 associated with multiple wedge fragments. (**b**) After open reduction and plate fixation, a cortical defect on the medial side was identified (black arrow). (**c**) A separate medial wedge fragment (white arrow) created a cortical defect and was at risk of devitalization if anatomical reduction had been attempted. (**d**) rhBMP-2 was applied to the medial cortical defect and along the fracture line between the stripped wedge fragment and the distal diaphysis (white arrow). (**e**) Postoperative radiograph showing fixation with a 3.5 mm locking plate and a 2.0 mm reduction plate. A hydroxyapatite (HA)-based carrier system containing 0.5 mg of rhBMP-2 is visible on the medial, lateral, and dorsal aspects of the ulnar fracture site. (**f**) Fracture healing was achieved without evidence of heterotopic ossification or radioulnar synostosis. The grafted material was resorbed during the fracture healing process. (**g**) The patient achieved a favorable functional outcome without any significant limitation in range of motion.

All patients underwent open reduction and internal fixation with plate or nail according to the types and characteristics of fractures (Figure 1). Fracture-related bone defects were managed by utilizing an rhBMP-2 delivery system (HA+rhBMP-2; NOVOSIS^®^, CGBio Inc., Seongnam, Republic of Korea), derived from *Escherichia coli* with an HA (Ca_10_(PO_4_)_6_(OH)_2_) carrier, which allows for slow controlled release in both inlay and onlay applications. The dosage was determined based on the defect size. The product was available in three formulations: 0.5 g of HA with 0.5 mg of rhBMP-2, 1.0 g of HA with 1.0 mg of rhBMP-2, and 3.0 g of HA with 3.0 mg of rhBMP-2. A maximum of 6 mg of rhBMP-2 was permitted per procedure. Patients received perioperative care consistent with the study protocol, including restrictions on medications that could potentially affect bone healing such as bisphosphonates, systemic steroids, and nonsteroidal anti-inflammatory drugs during the designated perioperative windows.

### 2.5. Statistical Analysis

All statistical analyses were planned prior to the study. There was no statistical hypothesis and the analyses were descriptive in nature. All statistical analyses were performed using IBM SPSS Statistics software, version 29.0.2.0 (IBM Corp., Armonk, NY, USA), with statistical significance set at *p* < 0.05. All participants who were treated at least once were included in the “full analysis set” (FAS; representing the intention-to-treat population). All participants with no major protocol deviations were included in the “per protocol set” (PPS). The FAS was the primary population for all efficacy and safety analyses. Missing efficacy data were handled using the last observation carried forward (LOCF) method, where applicable. Patient demographics and baseline characteristics-derived categorical variables were analyzed by descriptive statistics. Bone union and adverse event rates were calculated with two-sided 95% Confidence Intervals (CIs).

## 3. Results

**Figure 2:**
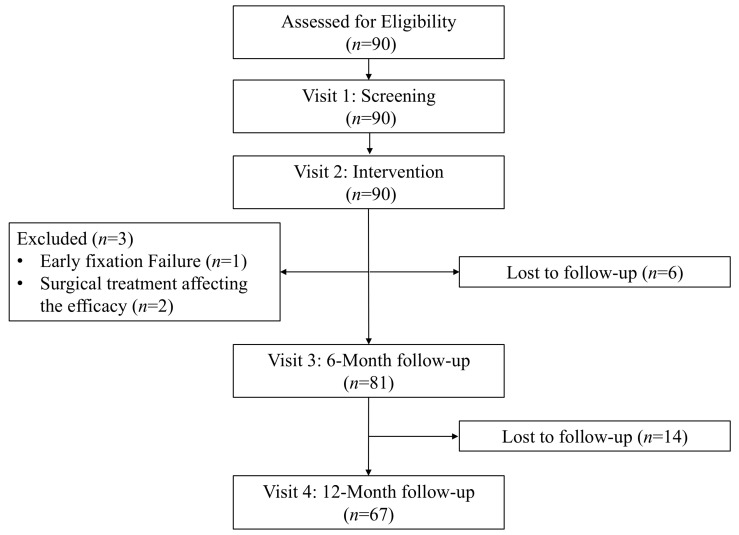
Flow chart of the study.

A total of 90 patients were initially enrolled in this multicenter study. Six patients were lost to follow-up at 6 months post-intervention, and three were excluded; one due to early fixation failure and two due to subsequent surgical treatments that could affect efficacy outcomes. Consequently, 81 patients with acute traumatic extremity fractures were included in the FAS. Issues of missing data for primary and secondary efficacy endpoints were addressed using the LOCF method, and all efficacy analyses were conducted based on the FAS population (Figure 2).

The mean age was 58.53 ± 19.18 years (range: 19–91), with 55.5% of the patients being men. The mean body mass index (BMI) was 24.9 ± 4.3 kg/m^2^. Most patients were non-smokers (75.3%), while 22.2% were current smokers. Comorbidities included hypertension (24.7%), diabetes mellitus (12.3%), cardiovascular disease (6.2%), and osteoporosis (2.5%). Fractures were more common in the upper extremity (60.5%) compared to the lower extremity (39.5%). Based on the Arbeitsgemeinschaft für Osteosynthesefragen/Orthopaedic Trauma Association (AO/OTA) classification, the occurrence of fracture types showed a distribution pattern of Type A (21.0%), Type B (34.6%), and Type C (44.4%). Open fractures were present in 18.6% of patients. Most fractures were treated using plate fixation (82.7%), followed by intramedullary nails (13.6%) and nail–plate combinations (3.7%). The mean time from injury to surgery was 1.13 ± 2.10 days. The mean dose of rhBMP-2 administered was 1.10 ± 1.26 mg. Among the patients, 39 (48.1%) received 0.5 mg, 34 (42.0%) received 1.0 mg, five (6.2%) received 3.0 mg, and three (3.7%) received 6.0 mg (Table 1).

In the FAS analysis using the LOCF method, the primary outcome of bone union at 12 months post-surgery was achieved in 77 of 81 patients (96.2%; 95% CI: 89.5–98.7%). At 6 months post-surgery, bone union was observed in 66 of 81 patients (81.5%; 95% CI: 71.7–88.4%). Treatment success, defined as bone union without additional surgical intervention, was achieved in 76 patients (95.0%; 95% CI: 87.8–98.0%). Only two patients (2.5%; 95% CI: 0.7–8.7%) required secondary surgical procedures for fracture healing (Table 2). Among the 15 patients classified as having non-union fractures at Visit 3, 10 achieved bone union by Visit 4 without requiring additional intervention. One patient, who underwent revisional internal fixation with autogenous bone grafting as a secondary intervention, also achieved bone union by Visit 4. In contrast, another patient who received the same secondary intervention remained in a non-union state at Visit 4. The remaining three patients did not undergo any additional intervention and remained in a state of non-union at Visit 4.

Adverse events were rare. At the 6-month follow-up, 80 of 81 patients (98.8%; 95% CI: 93.6–99.8%) exhibited no adverse events. A seroma was reported in one patient (1.2%; 95% CI: 0.2–6.4%). No adverse events were observed among the 67 patients assessed at the 12-month follow-up (Table 3). Two adverse events occurred following intervention. Patient AJ-15, diagnosed with a comminuted distal fibular fracture, developed a localized, mild seroma 1 month postoperatively. There was no clinical or microbiological evidence of infection. Causality was assessed as “possibly related,” and the patient received intravenous antibiotic therapy for two weeks. Patient KR-11, previously treated for osteomyelitis following a tibial shaft fracture, sustained a re-fracture and underwent plate removal with intramedullary nailing. A localized, mild infection developed 5 months postoperatively. The recurrence was suspected to be related to a pre-existing infection, with no evidence implicating rhBMP-2. Causality was determined to be unrelated to the study device. A staged operation was performed for fracture-related infection (Table 4). Therefore, patient KR-11 was excluded from the adverse event prevalence analysis due to the confounding influence of a prior infection. Cohen’s kappa values of the variables for inter-observer/intra-observer reliability were 0.76–0.88.

## 4. Discussion

This multicenter, prospective clinical trial is the first to evaluate the safety and efficacy of rhBMP-2 delivered via an HA carrier-based system, Novosis^TM^, in patients with acute fractures of the upper and lower extremities. Despite 18.6% of the cohort presenting with open fractures, the results demonstrated a notably high rate of radiographically validated bone union, which was 81.5% at 6 months and 96.2% at 12 months postoperatively. The treatment success rate, defined as bone union without the need for secondary surgical intervention, was 95.0%, highlighting robust functional efficacy in practical clinical application. Especially, at 6 months postoperatively, bone union was observed in 66 of 81 (81.5%) patients. These findings suggest a potentially positive role of rhBMP-2 in promoting fracture healing in the relatively early postoperative period when combined with an HA scaffold.

Conversely, five patients, three of whom had open fractures, did not achieve successful treatment, defined as bone union without the need for additional surgical intervention. These results highlight the need for individualized patient assessment and indicate that rhBMP-2 alone may not be sufficient in all cases, particularly in the presence of complex systemic or local factors inhibiting osteogenesis.

Adverse events reported during the study were minimal and non-severe. Only two adverse events of interest were recorded as follows: one localized seroma and one localized infection. Notably, the infection occurred in a patient with a history of osteomyelitis and was considered unrelated to the study device. The seroma resolved following conservative antibiotic therapy. These observations support the notion that HA-based rhBMP-2 delivery may mitigate some of the complications associated with previous carrier systems through sustained, controlled release, thereby reinforcing its safety profile. The absence of systemic complications, ectopic ossification, or severe inflammatory responses from the results of the current study may be attributed to the improved biocompatibility of the HA-based rhBMP-2 delivery system.

Several randomized prospective studies have assessed the efficacy and safety of rhBMP-2 in treating tibial fractures, with varying outcomes [4,14,15,41]. The Bone Morphogenetic Protein-2 Evaluation in Surgery for Tibial Trauma study on open tibial fractures treated with intramedullary nailing and routine soft-tissue management showed that the addition of rhBMP-2 (1.5 mg/mL) significantly reduced the need for secondary interventions, enhanced fracture and wound healing, and lowered infection rates compared with standard care alone [15]. Similarly, another study comparing rhBMP-2 combined with allograft to autogenous bone graft found comparable healing outcomes with reduced surgical morbidity [4]. However, other studies offer cautious conclusions. A randomized trial on open fractures treated with reamed nailing did not demonstrate significant improvement in healing and reported a trend toward higher infection rates [14]. Furthermore, a study utilizing a calcium phosphate matrix in closed fractures found no benefit in accelerating union or improving functional recovery [41]. Additionally, a multicenter randomized controlled trial comparing rhBMP-2 with allograft to autologous iliac crest bone graft in critical-sized tibial defects failed to demonstrate equivalence in union rates, while showing lower clinical healing, higher complication rates, and greater treatment costs in the rhBMP-2 group [17]. These findings suggest that while rhBMP-2 may be beneficial in specific high-risk fracture scenarios, its routine use in all tibial fractures is not supported by consistent evidence. Furthermore, while we aimed to evaluate efficacy across various anatomical sites, the limited sample size within specific subgroups, such as those based on age or specific fracture location, precluded a detailed statistical comparison with historical data for each category.

In the context of non-union fractures, BMP-2 also showed promising results. A retrospective review assessing its off-label use in non-union fractures demonstrated a 66% radiographically validated union rate following initial application [26]. Factors such as a smaller defect volume, partial cortical contact, and closed fracture pattern were associated with better outcomes. Despite these encouraging results, the high cost and inconsistent efficacy highlight the need for well-powered prospective trials to define the optimal role of BMP-2 in non-union fracture management. BMP-7 has shown comparable potential in promoting bone healing. Friedlaender et al. evaluated its use in tibial non-union fractures and reported fracture union rates exceeding 80% without additional bone grafting [23]. Giannoudis et al. demonstrated a synergistic effect when BMP-7 was combined with autografts in managing atrophic non-union fractures, proposing a biologically augmented approach to fracture healing [24]. Although these studies often utilized ACS carriers, the consistent efficacy observed across these studies underscores the fundamental role of BMP in osteo-induction.

Recent advancements in carrier systems have improved the efficacy of rhBMP-2 in managing critical-sized bone defects. A retrospective matched study using rhBMP-2 with an HA carrier in the induced membrane technique showed significantly increased bone density, enhanced corticalization under plates, and shorter time to union compared to controls [28]. Similarly, a prospective case series using rhBMP-2 with HA granules mixed with autologous bone achieved a 100% union rate within 1 year, without adverse effects or antibody formation [27]. These findings support the potential of advanced delivery systems to enhance the consistency, safety, and overall effectiveness of rhBMP-2 in bone regeneration.

Although rhBMP-2 has gained widespread acceptance, its clinical use has been tempered by safety concerns, particularly when delivered via collagen-based carriers. One large retrospective study documented increased rates of seroma formation, soft tissue swelling, and ectopic bone formation in trauma cases treated with rhBMP-2 [29]. A comprehensive review of adverse events attributed many of these complications to the burst-release kinetics of traditional collagen-based delivery systems, which result in supraphysiologic local concentrations of rhBMP-2. This rapid, unregulated release limits the duration of osteoinductive signaling and contributes to excessive soft tissue inflammation, ectopic ossification, and unintended tissue responses, underscoring the importance of optimizing both dosing and the delivery method [32].

HA-based carriers have emerged as a promising solution for delivering rhBMP-2. Unlike ACS, HA offers a structurally stable and biocompatible scaffold that enhances both osteoconduction and controlled protein release. Preclinical studies demonstrated that rhBMP-2 delivered via HA in calvarial and femoral models significantly promoted early bone regeneration with minimal inflammation and no ectopic ossification [33,34]. The sustained release profile of HA mitigates burst-release complications and helps maintain therapeutic levels of BMP-2 over time, enhancing both safety and efficacy. In the present study, adverse events were minimal, with only a single case of seroma observed, highlighting the excellent biocompatibility of HA. HA binds BMP-2 through interactions with functional groups (-OH, -NH_2_, and -COO-), as well as hydrogen bonding and Coulombic forces, allowing for controlled and prolonged release. This mechanism reduces early osteoclastogenesis activation and prevents subtherapeutic dosing during the later stages of healing [35,36,42,43]. In contrast, ACS are associated with BMP-2 leakage, poor structural integrity, and rapid resorption. HA offers improved handling characteristics and biological stability. Its micro- and macroporous architecture supports early bone ingrowth and provides strong osteoinductive properties. These properties contributed collectively to the favorable outcomes observed in this study.

Additionally, concerns regarding the potential tumorigenic effects of rhBMP-2 have been raised, particularly in the context of spinal fusion surgery, where high doses are often employed. Systematic reviews by Fu et al. [9] and Rodgers et al. [44] have reported an increased risk of cancer in patients treated with rhBMP-2. In the current study, no cases of malignancy were observed during the follow-up period. This may be attributed to the relatively lower doses used and the sustained-release properties of the HA carrier, which avoids the high-concentration burst release associated with collagen sponges. However, given the limited follow-up duration of 12 months, long-term monitoring is necessary to fully exclude potential carcinogenic risks.

The current study has several limitations. First, the single-arm design of the study precludes direct comparison with standard treatments such as autologous bone grafting or HA treatment alone, making it difficult to isolate the incremental benefit of rhBMP-2. A randomized controlled trial would provide stronger evidence for differences in efficacy and safety. Second, the study population exhibited significant heterogeneity regarding anatomical locations and fracture types. While this reflects real-world clinical practice, the relatively modest sample size limits the statistical power required to perform robust subgroup analyses or to draw definitive conclusions for specific fracture patterns. Third, the inclusion criteria were relatively broad, and the indication for rhBMP-2 use was subjective, relying on the clinical judgment of each surgeon to identify patients at high risk of non-union fractures. Additionally, the study involved three different surgeons from separate hospitals, which may have introduced selection bias.

Despite these limitations, a key strength of this study is its being the first multicenter, prospective clinical trial investigating the safety and efficacy of an HA carrier-based rhBMP-2 delivery system (Novosis^TM^) for the treatment of acute traumatic fractures of both the upper and lower extremities, including an FAS with the planned number of enrolled patients. Its design reflects real-world practice by including diverse fracture types, anatomical locations, and patient profiles. The integration of a modern HA carrier into the study design addresses previous limitations associated with collagen-based systems, offering sustained release and enhanced biocompatibility. A structured follow-up and standardized assessments enhance the reliability of the safety and efficacy data.

## 5. Conclusions

The HA-based rhBMP-2 application demonstrated favorable union rates and safety with minimal complications in treating acute upper and lower extremity fractures with bone defects. The controlled-release properties and biocompatibility of HA likely contributed to improved efficacy and reduced complications. Results should be interpreted as feasibility data from a heterogeneous case series without a control group. Future randomized controlled comparative trials with larger cohorts are warranted to validate efficacy and safety across various patient demographics and fracture types, as well as to establish clinical guidelines for optimal rhBMP-2 dosing and assess cost-effectiveness.

## Figures and Tables

**Table 1:** Patient demographics and baseline characteristics.

Variable	*N* (%)
Full Analysis Set	81
Age (years)	
Mean ± SD (Range)	58.53 ± 19.18 (19–91)
Sex	
Men	45 (55.5)
Women	36 (44.5)
BMI	
Mean ± SD (Range)	24.9 ± 4.3 (18.2–32.8)
Smoking Status	
Non-smoker	61 (75.3)
Former smoker	2 (2.5%)
Current smoker	18 (22.2)
Key Comorbidities	
Diabetes Mellitus	10 (12.3)
Hypertension	20 (24.7)
Osteoporosis	2 (2.5)
Cardiovascular disease	5 (6.2)
Fracture site	
Upper extremity	49 (60.5)
Lower extremity	32 (39.5)
Fracture site, Segment (AO/OTA)	
1 (Humerus or Clavicle)	18 (22.2)
2 (Radius or Ulna)	28 (34.6)
3 (Femur)	11 (13.6)
4 (Tibia)	17 (21.0)
7 (Metacarpal)	3 (3.7)
8 (Metatarsal)	4 (4.9)
Fracture type (AO/OTA)	
A	17 (21.0)
B	28 (34.6)
C	36 (44.4)
Open fracture (G-A classification)	
None	66 (81.4)
I	3 (3.7)
II	4 (4.9)
III	8 (9.9)
Fixation construct	
Plate	67 (82.7)
IM nail	11 (13.6)
Nail–plate combination	3 (3.7)
Dose of rhBMP-2 administered (mg)	
0.5	39 (48.1)
1.0	34 (42.0)
3.0	5 (6.2)
6.0	3 (3.7)
Time to surgery from injury (days)	
Mean ± SD (Range)	1.13 ± 2.10 (0–5)

AO/OTA, Arbeitsgemeinschaft für Osteosynthesefragen/Orthopaedic Trauma Association; BMI, body mass index; G-A, Gustilo-Anderson; IM, intramedullary; rhBMP-2, recombinant human Bone Morphogenetic Protein-2; SD, Standard deviation.

**Table 2:** Assessment of efficacy.

Primary Outcome	*N* (%), 95% Confidence Interval
Full-Set Analysis	81
Bone union (12 months)	77 (96.2), 89.5–98.7
Secondary Outcome	
Full-Set Analysis	81
Bone union (6 months)	66 (81.5), 71.7–88.4
The success rate of treatment	76 (95.0), 87.8–98.0
The incidence of additional surgical procedures	2 (2.5), 0.7–8.7

**Table 3:** Assessment of safety.

Prevalence of Adverse Events (Visit 3, 6 Months)	*N* (%), 95% Confidence Interval
Number of available	81
None	80 (98.8), 93.6–99.8
Seroma	1 (1.2), 0.2–6.4
Prevalence of Adverse Events (Visit 4, 12 months)	
Number of available	67
None	0 (0), 0.0–4.5

**Table 4:** Details of adverse events.

Patient Number	AJ-15	KR-11
AO/OTA classification	4F2C	42A
Applied Novosis concentration	0.5 mg	0.5 mg
Adverse events of interest	Seroma	Infection
Details of adverse event	No evidence of infection	Recurrence of infection suspected to relate to prior osteomyelitis with no relation to rhBMP-2 application
Time to adverse event	1 month	5 month
Fracture healing	Union (Visit 3)	Union (Visit 3)
Extent	Localized	Localized
Severity	Mild	Mild
Causality	Possibly	Not related
Secondary Intervention	Medication	Surgical treatment
Details of Secondary Intervention	IV antibiotics, 2 weeks	Staged surgical treatment for Fracture-Related Infection

AO/OTA, Arbeitsgemeinschaft für Osteosynthesefragen/Orthopaedic Trauma Association.

## Data Availability

The datasets generated during and/or analyzed during the current study are available from the corresponding author upon reasonable request.

## References

[1] Zura R., Xiong Z., Einhorn T., Watson J.T., Ostrum R.F., Prayson M.J., Della Rocca G.J., Mehta S., McKinley T., Wang Z. (2016). Epidemiology of fracture nonunion in 18 human bones. JAMA Surg..

[2] Rodham P.L., Giannoudis V.P., Kanakaris N.K., Giannoudis P.V. (2023). Biological aspects to enhance fracture healing. EFORT Open Rev..

[3] Mauffrey C., Barlow B.T., Smith W. (2015). Management of segmental bone defects. J. Am. Acad. Orthop. Surg..

[4] Jones A.L., Bucholz R.W., Bosse M.J., Mirza S.K., Lyon T.R., Webb L.X., Pollak A.N., Golden J.D., Valentin-Opran A., BMP-2 Evaluation in Surgery for Tibial Trauma-Allgraft (BESTT-ALL) Study Group (2006). Recombinant human BMP-2 and allograft compared with autogenous bone graft for reconstruction of diaphyseal tibial fractures with cortical defects. A randomized, controlled trial. J. Bone Jt. Surg. Am..

[5] Kim H., Kar A.K., Kaja A., Lim E.J., Choi W., Son W.S., Oh J.K., Sakong S., Cho J.W. (2021). More weighted cancellous bone can be harvested from the proximal tibia with less donor site pain than anterior iliac crest corticocancellous bone harvesting: Retrospective review. J. Orthop. Surg. Res..

[6] Valentin-Opran A., Wozney J., Csimma C., Lilly L., Riedel G.E. (2002). Clinical evaluation of recombinant human bone morphogenetic protein-2. Clin. Orthop. Relat. Res..

[7] McKay W.F., Peckham S.M., Badura J.M. (2007). A comprehensive clinical review of recombinant human bone morphogenetic protein-2 (INFUSE^®^ Bone Graft). Int. Orthop..

[8] Valdes M.A., Thakur N.A., Namdari S., Ciombor D.M., Palumbo M. (2009). Recombinant bone morphogenetic protein-2 in orthopaedic surgery: A review. Arch. Orthop. Trauma Surg..

[9] Fu R., Selph S., McDonagh M., Peterson K., Tiwari A., Chou R., Helfand M. (2013). Effectiveness and harms of recombinant human bone morphogenetic protein-2 in spine fusion: A systematic review and meta-analysis. Ann. Intern. Med..

[10] Gillman C.E., Jayasuriya A.C. (2021). FDA-approved bone grafts and bone graft substitute devices in bone regeneration. Mater. Sci. Eng. C Mater. Biol. Appl..

[11] Cheng H., Jiang W., Phillips F.M., Haydon R.C., Peng Y., Zhou L., Luu H.H., An N., Breyer B., Vanichakarn P. (2003). Osteogenic activity of the fourteen types of human bone morphogenetic proteins (BMPs). J. Bone Jt. Surg. Am..

[12] Kingsley D.M. (1994). The TGF-beta superfamily: New members, new receptors, and new genetic tests of function in different organisms. Genes. Dev..

[13] Alt V., Borgman B., Eicher A., Heiss C., Kanakaris N.K., Giannoudis P.V., Song F. (2015). Effects of recombinant human Bone Morphogenetic Protein-2 (rhBMP-2) in grade III open tibia fractures treated with unreamed nails—A clinical and health-economic analysis. Injury.

[14] Aro H.T., Govender S., Patel A.D., Hernigou P., Perera de Gregorio A., Popescu G.I., Golden J.D., Christensen J., Valentin A. (2011). Recombinant human bone morphogenetic protein-2: A randomized trial in open tibial fractures treated with reamed nail fixation. J. Bone Jt. Surg. Am..

[15] Govender S., Csimma C., Genant H.K., Valentin-Opran A., Amit Y., Arbel R., Aro H., Atar D., Bishay M., Börner M.G. (2002). Recombinant human bone morphogenetic protein-2 for treatment of open tibial fractures: A prospective, controlled, randomized study of four hundred and fifty patients. J. Bone Jt. Surg. Am..

[16] Krause F., Younger A., Weber M. (2008). Recombinant human BMP-2 and allograft compared with autogenous bone graft for reconstruction of diaphyseal tibial fractures with cortical defects. J. Bone Jt. Surg. Am..

[17] Major Extremity Trauma Research Consortium (METRC) (2019). A randomized controlled trial comparing rhBMP-2/Absorbable collagen sponge versus autograft for the treatment of tibia fractures with critical size defects. J. Orthop. Trauma.

[18] Starr A.J. (2003). Recombinant human bone morphogenetic protein-2 for treatment of open tibial fractures. J. Bone Jt. Surg. Am..

[19] Bilic R., Simic P., Jelic M., Stern-Padovan R., Dodig D., van Meerdervoort H.P., Martinovic S., Ivankovic D., Pecina M., Vukicevic S. (2006). Osteogenic protein-1 (BMP-7) accelerates healing of scaphoid non-union with proximal pole sclerosis. Int. Orthop..

[20] Caterini R., Potenza V., Ippolito E., Farsetti P. (2016). Treatment of recalcitrant atrophic non-union of the humeral shaft with BMP-7, autologous bone graft and hydroxyapatite pellets. Injury.

[21] Courvoisier A., Sailhan F., Laffenêtre O., Obert L., French Study Group of BMP in Orthopedic Surgery (2014). Bone morphogenetic protein and orthopaedic surgery: Can we legitimate its off-label use?. Int. Orthop..

[22] Crawford C.H., Seligson D. (2009). Atrophic nonunion of humeral diaphysis treated with locking plate and recombinant bone morphogenetic protein: Nine cases. Am. J. Orthop..

[23] Friedlaender G.E., Perry C.R., Cole J.D., Cook S.D., Cierny G., Muschler G.F., Zych G.A., Calhoun J.H., LaForte A.J., Yin S. (2001). Osteogenic protein-1 (bone morphogenetic protein-7) in the treatment of tibial nonunions. J. Bone Jt. Surg. Am..

[24] Giannoudis P.V., Kanakaris N.K., Dimitriou R., Gill I., Kolimarala V., Montgomery R.J. (2009). The synergistic effect of autograft and BMP-7 in the treatment of atrophic nonunions. Clin. Orthop. Relat. Res..

[25] Pecina M., Haspl M., Jelic M., Vukicevic S. (2003). Repair of a resistant tibial non-union with a recombinant bone morphogenetic protein-7 (rh-BMP-7). Int. Orthop..

[26] Starman J.S., Bosse M.J., Cates C.A., Norton H.J. (2012). Recombinant human bone morphogenetic protein-2 use in the off-label treatment of nonunions and acute fractures: A retrospective review. J. Trauma Acute Care Surg..

[27] Choi W., Kim B.S., Cho W.T., Lim E.J., Choi J.S., Ryu Y.K., Cho J.W., Sakong S., Oh J.K. (2024). Efficacy and safety of recombinant human bone morphogenetic protein-2 (rhBMP-2) combined with autologous bone for the treatment of long bone nonunion: A report of a prospective case series. Injury.

[28] Son W.S., Lim E.J., Sakong S., Kim H., Choi W., Cho J.W., Oh J.K. (2022). Effect of recombinant human bone morphogenetic Protein-2 (rhBMP-2) with hydroxyapatite carrier in induced membrane technique: A retrospective propensity score-matched study. J. Orthop. Trauma.

[29] Chan D.S., Garland J., Infante A., Sanders R.W., Sagi H.C. (2014). Wound complications associated with bone morphogenetic protein-2 in orthopaedic trauma surgery. J. Orthop. Trauma.

[30] Garg S., McCarthy J.J., Goodwin R., Kolmodin J., Sankar W.N., Franklin C., Armstrong D., Fryzel D., Hassenbein S., Meder C. (2017). Complication rates after bone morphogenetic protein (BMP) use in orthopaedic surgery in children: A concise multicenter retrospective cohort study. J. Pediatr. Orthop..

[31] Agrawal V., Sinha M. (2017). A review on carrier systems for bone morphogenetic protein-2. J. Biomed. Mater. Res. B Appl. Biomater..

[32] James A.W., LaChaud G., Shen J., Asatrian G., Nguyen V., Zhang X., Ting K., Soo C. (2016). A review of the clinical side effects of bone morphogenetic Protein-2. Tissue Eng. Part B Rev..

[33] Chung C.H., Kim Y.K., Lee J.S., Jung U.W., Pang E.K., Choi S.H. (2015). Rapid bone regeneration by *Escherichia coli*-derived recombinant human bone morphogenetic protein-2 loaded on a hydroxyapatite carrier in the rabbit calvarial defect model. Biomater. Res..

[34] Dang L.H.N., Kim Y.K., Kim S.Y., Lim K.J., Bode K., Lee M.H., Lee K.B. (2019). Radiographic and histologic effects of bone morphogenetic protein-2/hydroxyapatite within bioabsorbable magnesium screws in a rabbit model. J. Orthop. Surg. Res..

[35] Dong X., Wang Q., Wu T., Pan H. (2007). Understanding adsorption-desorption dynamics of BMP-2 on hydroxyapatite (001) surface. Biophys. J..

[36] Yun P.Y., Kim Y.K., Jeong K.I., Park J.C., Choi Y.J. (2014). Influence of bone morphogenetic protein and proportion of hydroxyapatite on new bone formation in biphasic calcium phosphate graft: Two pilot studies in animal bony defect model. J. Craniomaxillofac Surg..

[37] Templeman D.C., Gulli B., Tsukayama D.T., Gustilo R.B. (1998). Update on the management of open fractures of the tibial shaft. Clin. Orthop. Relat. Res..

[38] Watson J.T., Anders M., Moed B.R. (1995). Management strategies for bone loss in tibial shaft fractures. Clin. Orthop. Relat. Res..

[39] Litrenta J., Tornetta P., Mehta S., Jones C., O’Toole R.V., Bhandari M., Kottmeier S., Ostrum R., Egol K., Ricci W. (2015). Determination of Radiographic Healing: An Assessment of Consistency Using RUST and Modified RUST in Metadiaphyseal Fractures. J. Orthop. Trauma.

[40] Metsemakers W.J., Morgenstern M., McNally M.A., Moriarty T.F., McFadyen I., Scarborough M., Athanasou N.A., Ochsner P.E., Kuehl R., Raschke M. (2018). Fracture-related infection: A consensus on definition from an international expert group. Injury.

[41] Lyon T., Scheele W., Bhandari M., Koval K.J., Sanchez E.G., Christensen J., Valentin A., Huard F. (2013). Efficacy and safety of recombinant human bone morphogenetic protein-2/calcium phosphate matrix for closed tibial diaphyseal fracture: A double-blind, randomized, controlled phase-II/III trial. J. Bone Jt. Surg. Am..

[42] Kang W., Lee D.S., Jang J.H. (2015). Evaluation of sustained BMP-2 release profiles using a novel fluorescence-based retention assay. PLoS ONE.

[43] Cho J.H., Song H.K. (2025). Current concepts and applications of bone graft substitutes in orthopedic surgery. J. Musculoskelet. Trauma.

[44] Rodgers M.A., Brown J.V., Heirs M.K., Higgins J.P., Mannion R.J., Simmonds M.C., Stewart L.A. (2013). Reporting of industry-funded study outcome data: Comparison of confidential and published data on the safety and effectiveness of rhBMP-2 for spinal fusion. BMJ.

